# Professional perspectives on PN among registered dietitians in Saudi Arabia: a mixed-methods assessment

**DOI:** 10.3389/fnut.2026.1695919

**Published:** 2026-03-23

**Authors:** Yasmin Algindan, Hoda AboAlsamh, Shabir Ahmad

**Affiliations:** 1 Department of Clinical Nutrition, College of Applied Medical Sciences, Imam Abdulrahman bin Faisal University, Dammam, Saudi Arabia; 2 College of Business Administration, Imam Abdulrahman bin Faisal University, Dammam, Saudi Arabia; 3 College of Business, Al Yamamah University, Al Khobar, Saudi Arabia

**Keywords:** multi-omics, omics-technology, personalized nutrition, PN, registered dietitians, Saudi Arabia

## Abstract

**Background:**

The clinical implementation of personalized nutrition (PN) remains limited, despite growing interest. Healthcare professionals’ readiness for adoption must be better understood.

**Objective:**

The aim of this study is to explore registered dietitians’ perceptions, attitudes, and readiness regarding PN and multi-omics approaches in Saudi Arabia.

**Method:**

A mixed-methods survey of 88 registered dietitians in Saudi Arabia provided quantitative and qualitative data on personalized nutrition technology (PNT) adoption patterns and barriers, analysed using the Consolidated Framework for Implementation Research (CFIR) to identify implementation barriers and facilitators.

**Results:**

PNT adoption was low (16%). Work experience, but not education level, had a relatively greater correlation with perceived usefulness of PNT (*r* = 0.280, *p* = 0.007). Genomics, metabolomics, and microbiome testing demonstrated strong correlations with PNT usefulness (*r* = 0.321–0.571, *p* ≤ 0.004). Qualitative findings identified knowledge gaps as the primary challenge and emphasized education as the key facilitator.

**Conclusion:**

While Saudi dietitians have positive attitudes toward PN, they face significant implementation barriers at both an organizational and educational levels. For PN integration to be achieved within the Kingdom’s healthcare system, policy, academic, and institutional interventions are required to enhance organizational support, professional training gaps, and interprofessional collaboration frameworks.

## 1 Introduction

Nutritional disorders have become a major problem worldwide, requiring more targeted interventions. At present, dietary advice is quite broad and is directed towards the entire population without taking into account factors like genetic, environmental, and cultural differences. They give one-size-fits-all advice to address chronic disease risk factors, such as obesity and type II diabetes. The impact has been minimal until now. Diverse individual responses to interventions have been observed, supporting the need to develop strategies based on delivering more personalized advice to individuals (1).

The growing epidemic of nutrition-related diseases in Saudi Arabia, including 39.8% metabolic syndrome, 23.1% obesity, and a projected top-5 global ranking by 2030 for type 2 diabetes (2, 3, 4), demonstrates the limitations of standardized diets. As obesity has an annual societal cost of $116.85 billion (5), PN interventions that take into account individual genetic, metabolic, and behavioural variances are imperative in preventing and managing the disease.

At a broader level, the high incidence of metabolic syndrome and diet-related diseases in the Middle East and North Africa (MENA) region is closely linked to unhealthy dietary patterns. Due to historical migrations, consanguinity, and geographical factors, populations in the MENA region have distinct genetic makeups. As a result of this genetic distinction, nutritional studies conducted on European or Western populations may not be directly relevant (6) As well as being a significant underrepresented population in public genetic databases, this region is prone to genetic diseases and significant health challenges.

A recent review indicates that six population genome initiatives are presently being conducted in the MENA region, specifically in the Kingdom of Saudi Arabia, Qatar, Egypt, the United Arab Emirates, Bahrain, and Iran. MENA is rapidly increasing its involvement in national-scale genomic data collection, which will lead to increased representation in public genetic databases soon. A key point of the review was that ethical and regulated genomic initiatives can enhance patient prognosis and quality of life by enabling development of PN treatments (7).

With advances in multi-omic sciences over the past few decades, PN has emerged as an innovative way to provide nutritional guidance based on individual genetic profiles, aiming to customize nutritional intake and functional food consumption. The goal of this concept is to prevent chronic diet-related diseases through the integration of healthcare strategies that go beyond universal dietary recommendations (8). By 2032, the global market for PN is expected to reach $50 billion (9). The market is driven by noncommunicable disease prevalence, advancing omics technologies, and commercial platforms that offer genetic and biomarker-based dietary services (10–12).

In a recent thesis, AI and machine learning have demonstrated the potential to generate culturally-adapted dietary recommendations for Arab populations by integrating traditional food preferences with individual health data (13). Despite the region’s growing genomic databases and healthcare transformation goals, AI-driven PN research remains scarce in the Middle East.

Underlying Saudi Arabia’s ambitious national transformation guided by Vision 2030, is a robust technology and innovation framework (14). To advance the field of PN in the Kingdom, this national framework, particularly its National Strategy for Research, Development, and Innovation (RDIA), can be applied (15). Health and Well-being, as well as Economies of the Future, are two of the four pillars of Saudi Arabia’s RDIA Strategies that can be aligned with PN. Technological sophistication required for PN fits perfectly into the “Economies of the Future” pillar. One of the first and only PN programs in Saudi Arabia, NUTREOM, is a demonstration of the intention to integrate cutting-edge health technology into future Saudi cities (16).

In the Middle East, PN has emerged as a solution to the high rate of non-communicable diseases and to the digital transformation agenda. These initiatives combine government programs, specialized clinical services, and technology-driven startups. Regional examples of PN initiatives in United Arab Emirates; Personalised Weight Management Programme In Abu Dhabi (17), Nutrigenomics Services at ADSCC (18), Nutrigenomix (19).

Yet, PN is still early in Saudi Arabia. Though government Vision 2030 initiatives, increased chronic disease prevalence, and rising standards of living create significant potential, their implementation requires research and investigation tailored to Saudi culture to overcome challenges. This study aims to identify gaps, requirements, and the readiness of dietitians in Saudi Arabia to adopt PN from a dietetics perspective.

## 2 Methods

### Questionnaire

In this mixed method study, the researcher employed a validated questionnaire to collect data. The structure and content of the questionnaire were derived from a previously published work by Abrahams et al. (20), which provides detailed information about the instrument’s development, validation, and sections. To ensure proper usage and adherence to ethical guidelines, the authors (20) of the original study were contacted via email. Approval of the use of the questionnaire was obtained on the condition that their published paper will be acknowledged.

The Institutional Review Board at Imam Abdulrahman bin Fasal University granted ethical approval (Approval Number: IRB-2024-03-372). An information sheet explaining the study’s purpose, procedures, and participants’ rights was provided before the survey began.

The questionnaire consists of 62 questions, with an estimated completion time ranging from 15 to 20 min. It is organized into distinct sections, each addressing specific aspects related to the study’s objectives and variables of interest, as outlined in the accompanying table.

In addition to the validated questionnaire, the researchers aimed to tailor this study to Saudi Arabian practices. Three questions were added at the end of the survey to assess the gaps and requirements specific to Saudi Arabia. The additional questions are as follows:

- What do you perceive as the main gaps or barriers in PN practices adoption in Saudi Arabia?
- Based on your experience, what elements or facilitators are essential for the implementation of PN strategies in Saudi Arabia?
- In view of the unique cultural, dietary, and social context of Saudi Arabia, what are your thoughts on the most critical areas to be addressed to develop and implement nutritional strategies tailored to the Saudi population?”

By incorporating these open-ended questions, qualitative data complemented the validated questionnaire findings. The responses to these questions provided a deeper understanding of the specific gaps, requirements, and considerations relevant to the successful implementation of PN in Saudi Arabia.

As a key component of the survey items, we used the term ‘PN’ as an inclusive term that encompassed technologies across the PN spectrum. Participants were not provided with restrictive definitions, allowing their responses to reflect their professional context. In Saudi healthcare literature, there is little standardization of PN terminology, there is wide variation in technology access among healthcare facilities, and the research objective is to assess readiness for PN implementation broadly rather than for specific omics applications. Further interview questions explored which technologies and approaches participants associated with PN.

### Sampling

With an *α* of 0.05, a power of 0.9, and an anticipated effect size of 0.8, a total sample size of 88 participants was deemed necessary for the study. Dietitians were accessed through dietetics associations, universities, hospitals, and dietetics-related social media networks.

Inclusion criteria: SCHS registered dietitians working in hospitals, clinics, or as freelancers, working in Saudi Arabia.

Exclusion criteria: Dietitians with no SCHS registration or not working in Saudi Arabia.

### Research questions

- What are the attitudes and perceptions of registered dietitians toward the clinical utility and effectiveness of PN technologies?
- How do demographic and professional characteristics (experience, education, work setting) influence dietitians’ readiness for PN adoption?
- To what extent are registered dietitians in Saudi Arabia ready to adopt and integrate PN approaches into their clinical practice?
- What are the infrastructure and resource gaps at the community and organizational levels that need to be addressed for successful PN adoption?

### Study design and data collection

The online survey consisted of both closed-ended and open-ended questions, enabling the collection of quantitative and qualitative data. A total of 88 respondents participated in the study.

#### Quantitative data analysis

The quantitative component of the survey included Likert-scale and multiple-choice items designed to assess knowledge, attitudes, and perceived barriers and facilitators related to PN. Descriptive statistics (means, standard deviations, and frequencies) were calculated to summarize participants’ responses. Inferential analyses, including independent samples *t*-tests, were conducted to examine differences across demographic of PN adopters and non-adopters’ groups. Independent samples *t*-tests were selected to compare demographic and professional characteristics between PN adopters and non-adopters, as the grouping variable consisted of two independent categories and the outcome variables were treated as approximately continuous. Prior to analysis, assumptions of independence and approximate normality were considered acceptable given the study design and sample characteristics. Correlation and regression-based analyses were employed to examine associations between professional attributes and perceived usefulness of PN technologies, as these tests are appropriate for assessing the strength and direction of relationships in cross-sectional survey data. Quantitative analysis was performed using IBM SPSS Statistics.

#### Qualitative data analysis

The final section of the survey included three open-ended questions designed to elicit participants’ views on the barriers, facilitators, and culturally specific considerations related to implementing PN practices in Saudi Arabia. Responses were analysed using inductive qualitative content analysis to identify emerging patterns and themes. The textual data were imported into NVivo, which facilitated systematic coding, theme development, and data organization.

The analysis process involved multiple readings of the responses to ensure familiarity with the content, followed by initial open coding to capture key concepts. Codes were then grouped into categories, which were further refined into overarching themes that reflected shared experiences and insights among the respondents. This approach allowed for the identification of recurrent ideas, nuanced perspectives, and contextual factors specific to the Saudi Arabian setting. To enhance reliability, a subset of responses was independently coded by two researchers, with discrepancies resolved through discussion to ensure consistency in interpretation.

To enhance the rigor of the qualitative analysis, two independent researchers coded the open-ended responses using an inductive thematic approach. The coders first reviewed a subset of responses to refine the coding framework and then independently coded the full dataset. Inter-rater reliability was assessed using Cohen’s kappa, which indicated substantial agreement (*κ* = 0.78). Discrepancies were resolved through discussion until consensus was achieved. Data saturation was considered reached when no new themes emerged after repeated review of the responses; the final cycles of coding yielded only repetitions of previously identified concepts, indicating that the thematic structure was sufficiently comprehensive.

## 3 Results

### Quantitative results

#### Demographic profile of respondent

The demographic profile of the participants (*N* = 88) presented in Table 1 revealed that a significant majority were women (83%), with men representing only 17% of the sample. Most participants (registered dietitians) were either in the early career age group (23–29 years), which accounted for 41%, or the emerging professionals’ group (30–39 years), comprising 34% of the sample. Mid-career participants aged 40–49 years represented 19%, and senior professionals aged 50 and above accounted for 6%. Adoption of PN technologies (PNT) was relatively low, with only 16% (*n* = 14) of respondents identifying as adopters, compared to 84% non-adopters. This low number of PNT adopters limits the robustness of subgroup comparisons and reduces statistical power for detecting between-group differences. Similarly, although correlation and regression analyses were conducted, some path coefficients may be underpowered due to sample size constraints. These findings should therefore be interpreted cautiously and viewed as exploratory rather than confirmatory.

**Table 1:** Demographic statistics.

Demographic variables	Categories	Frequency	Percent
Gender	Man	15	17%
	Woman	73	83%
Age	Early career (23–29 years)	36	41%
	Emerging professionals (30–39 years)	30	34%
	Mid-career (40–49 years)	17	19%
	Senior professionals (50 + years)	5	6%
PN technologies adoption	Adopters	14	16%
	No-adopters	74	84%
Work experience	Entry-level (0–4 years)	30	34%
	Early career (5–9 years)	27	31%
	Developing professional (10–14 years)	11	13%
	Mid-career (15–19 years)	10	11%
	Senior/veteran (20 + years)	10	11%
Qualification	BSc	41	47%
	Masters	28	32%
	Postgraduate diploma	2	2%
	PhD/Mphil by thesis	12	14%
	Doctorate	5	6%
Sector	Non-profit/charity	5	6%
	Not clinical	5	6%
	Private	32	36%
	Public	46	52%
City	Abha	1	1%
	Alqassim	2	2%
	Dammam	20	23%
	Dhahran	2	2%
	Hail	1	1%
	Jeddah	10	11%
	Jizan	1	1%
	Jubail	3	3%
	Khobar	10	11%
	Mecca	2	2%
	Medina	3	3%
	Qatif	2	2%
	Riyadh	29	33%
	Tabuk	1	1%
	Taif	1	1%
		*N* = 88	

In terms of qualifications, 47% held a bachelor’s degree, 32% held a master’s degree, and 20% had postgraduate or doctoral-level qualifications. Most participants worked in the public sector (52%), followed by the private sector (36%), with smaller proportions in the nonprofit and nonclinical sectors. Geographically, respondents were distributed across various cities in Saudi Arabia, with the highest concentration in Riyadh (33%), followed by Dammam (23%), and Jeddah and Khobar (11% each).

To explore whether demographic or professional background characteristics differed between registered dietitians who adopt PN (PN) technologies and those who do not, independent samples *t*-tests were conducted. This comparison helps determine whether adoption patterns may be influenced by underlying group differences. Table 2 shows that no statistically significant differences emerged across any of the variables assessed. Gender, age, and work experience displayed similar mean scores between adopters and non-adopters, with *p*-values ranging from 0.478 to 0.487. Likewise, qualification level and employment sector did not differ meaningfully between the groups (*p* = 0.455 and *p* = 0.609, respectively). Overall, the results suggest that PN adoption in this sample is not associated with variations in demographic or professional characteristics.

**Table 2:** Differences between PN adopters and non-adopters nutrigenetic testing into their practice.

Variables	PN adopters (*N* = 14)		PN non-adopters (*N* = 74)		Independent samples *t*-test		
	Mean	SD	Mean	SD	*t* value	Df	*p* value
Gender	1.714	0.452	1.851	0.356	−1.073	1.509	0.478
Age	1.714	0.881	1.932	0.905	−0.845	2.562	0.487
Work experience	2.071	1.334	2.405	1.345	−0.858	2.545	0.481
Qualification	2.286	1.278	1.946	1.229	0.918	2.322	0.455
Sector	2.714	0.7	2.851	0.456	−0.705	1.064	0.609

#### Work experience, education, and PNT perceived usefulness

The analysis in Table 3 shows that work experience has a statistically significant correlation with the perceived usefulness of PN technologies (PNT). The correlation coefficient is 0.282, and the Holm-adjusted *p*-value of 0.028 confirms a meaningful positive association at the 95% confidence level. As shown in Table 3, work experience demonstrated a statistically significant bivariate association with perceived usefulness of PN technologies (*r* = 0.282, Holm-adjusted *p* = 0.028), indicating that greater professional experience is associated with more favourable perceptions of PNT. However, when examined using adjusted path coefficients, this association was no longer statistically significant (*β* = 0.263, Holm-adjusted *p* = 0.063). This distinction suggests that while work experience is correlated with perceived usefulness at the bivariate level, it does not retain statistical significance after adjustment and therefore should not be interpreted as a predictive or independent effect.

**Table 3:** Qualification and work experience relationship to perceived usefulness of PNT.

Variables	Correlation coefficient					
	*r*	SD	*t*-value	*p*-values	Holm-adjusted *p*	Decision
Academic qualification	0.142	0.109	1.277	0.202	0.404	Insignificant
Work experience	0.282	0.104	2.689	0.007	0.028	Significant

Variables	Path coefficient					
	*β*	SD	*t*-value	*p*-values	Holm-adjusted *p*	Decision
Academic qualification	0.061	0.114	0.519	0.604	0.404	Insignificant
Work experience	0.263	0.113	2.314	0.021	0.063	Insignificant

*r*, correlation coefficient; SD, standard deviation; *β*, path coefficient; significance @ 95%, two tailed (*t* ≥ ±1.96; *p* < 0.05).

However, academic qualification showed no significant association with perceived usefulness in either bivariate or adjusted analyses, indicating that formal education level does not appear to be related to dietitians’ perceptions of PNT usefulness. All quantitative findings are reported as associations derived from cross-sectional data and do not imply causality or predictive relationships.

Given the sample size, particularly the small subgroup of PN adopters, these associations should be interpreted with caution, and non-significant path coefficients may reflect limited statistical power rather than the absence of meaningful relationships.

#### Areas of importance for registered dietitians correlating with perceived usefulness of PNT

To examine which areas of professional interest are associated with registered dietitians’ perceived usefulness of PN technologies (PNT), Pearson correlation coefficients were calculated between PNT usefulness and the rated importance of several emerging and traditional domains within dietetic practice (Table 4). The results reveal several noteworthy patterns.

**Table 4:** PNT usefulness correlation with areas of importance.

Areas of importance	*r*	SD	*t*-value	*p*-values	Holm-adjusted *p*	Decision
Business marketing	−0.057	0.102	0.558	0.577	1.864	Insignificant
Artificial intelligence and machine learning	0.179	0.114	1.570	0.117	0.936	Insignificant
Bioinformatics	0.086	0.118	0.729	0.466	1.864	Insignificant
Chatbots	0.172	0.120	1.429	0.153	0.936	Insignificant
Creativity, innovation, and entrepreneurship	0.259	0.110	2.352	0.019	0.209	Insignificant
Food engineering	−0.007	0.116	0.058	0.954	1.864	Insignificant
Functional integrative nutrition	0.221	0.124	1.775	0.076	0.684	Insignificant
Genomics	0.321	0.111	2.882	0.004	0.050	Significant
Metabolomics	0.571	0.091	6.290	0.000	0.002	Significant
Microbiome testing	0.438	0.106	4.140	0.000	0.002	Significant
Research	0.213	0.106	2.022	0.043	0.430	Insignificant
Teaching and training	0.182	0.116	1.567	0.117	0.936	Insignificant
Telehealth	0.064	0.146	0.442	0.659	1.864	Insignificant
Virtual augmented (VR) reality	0.287	0.103	2.791	0.005	0.060	Insignificant
Wearable technology	0.204	0.148	1.380	0.168	0.936	Insignificant

*r*, correlation coefficient; SD, standard deviation; significance @ 95%, two tailed (*t* ≥ ±1.96; *p* < 0.05).

The analysis (Table 4) shows that only a few areas retained statistically significant associations with the perceived usefulness of PNT after applying Holm–Bonferroni corrections. Metabolomics demonstrated the strongest positive correlation (*r* = 0.571, adjusted *p* = 0.002), indicating that dietitians who value metabolic profiling are substantially more inclined to view PNT as useful in supporting individualized dietary recommendations. Microbiome testing also showed a robust significant relationship (*r* = 0.438, adjusted *p* = 0.002), reflecting the growing recognition of gut microbiome insights as a core component of PN. In addition, genomics remained significant following adjustment (*r* = 0.321, adjusted *p* = 0.050), highlighting the continued importance of genetic information in shaping individualized nutrition plans.

Several other areas initially showed meaningful unadjusted associations but did not remain significant after correction. These included creativity, innovation, and entrepreneurship, virtual and augmented reality, research, and functional integrative. Although no longer statistically significant, the positive direction of these relationships suggests that practitioners who value innovation, emerging technologies, and evidence-based practice tend to perceive PNT more favourably.

Overall, the findings indicate that support for PNT is strongest among dietitians who prioritize biologically grounded, science-driven domains, particularly metabolomics, microbiome testing, and genomics, which represent key pillars in the advancement of PN.

#### Perceived usefulness of PNT in relation to the importance placed on microbiome testing and metabolomics

The analysis shows a strong positive correlation between PNT usefulness and the perceived importance of metabolomics (*r* = 0.571, *p* < 0.001), the highest among all measured variables. Similarly, a significant positive correlation was found with microbiome testing (*r* = 0.438, *p* < 0.001),

#### Percentage of organizations actively promoting creativity and innovation

**Figure 1:**
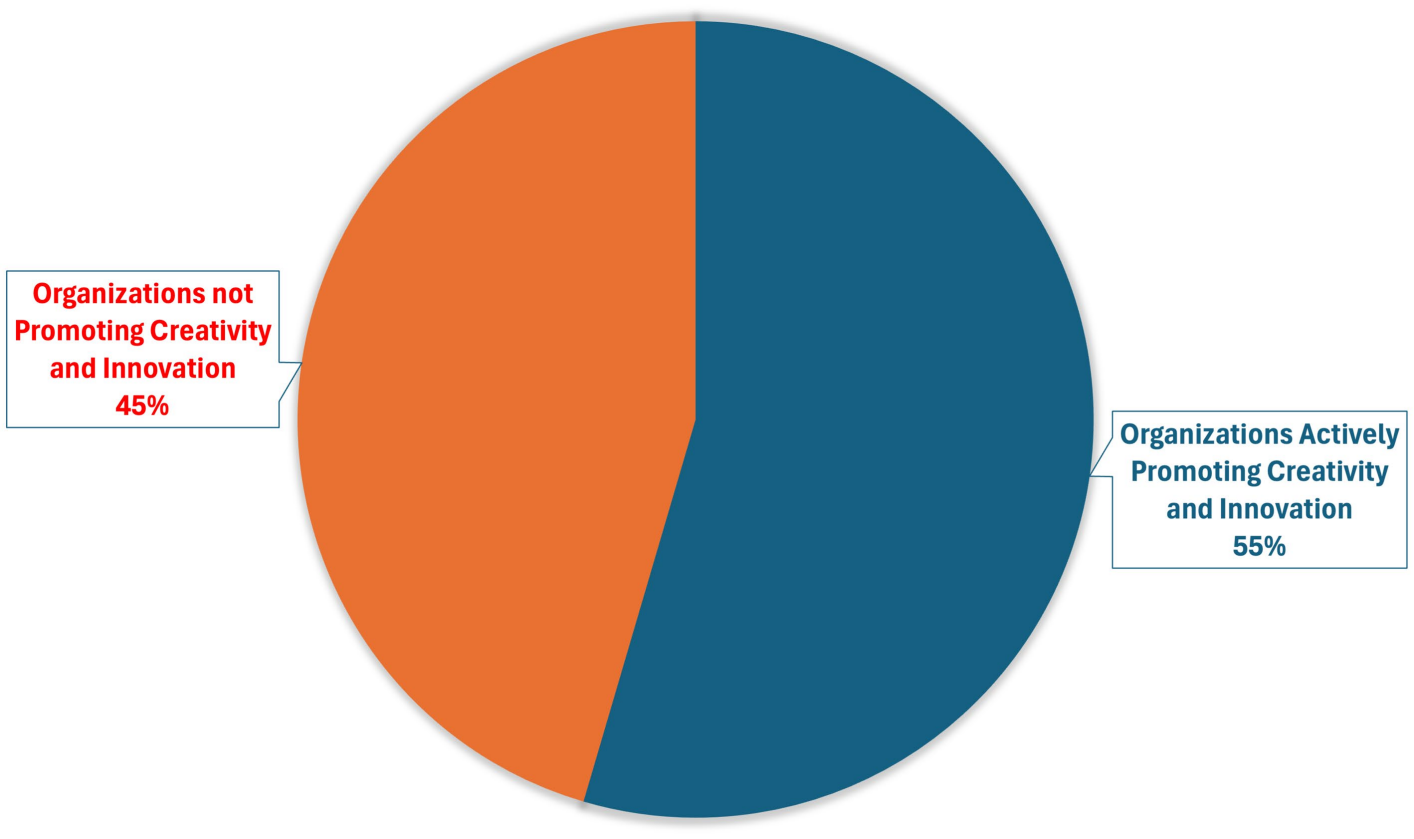
Organizational promotion of creativity and innovation.

To assess perceptions of organizational support for creativity and innovation, registered dietitians (RDs) were asked to indicate their level of agreement with the statement “Organizations actively promote creativity and innovation,” using a five-point Likert scale (1 = completely disagree to 5 = completely agree). Out of 88 respondents, 48 RDs (54.5%) reported either agreeing or completely agreeing with the statement, see Figure 1.

#### Public vs. private-sector PN adopters and their qualifications

To explore whether PN adopters are more likely to come from specific sectors and to understand their qualification levels, descriptive statistics were analysed (Table 5). Half of the PN adopters (50%) were from the public sector, followed by 36% from the private sector and 14% from non-profit or charity organizations. Regarding educational qualifications, most adopters held either a bachelor’s degree (36%) or a master’s degree (29%), while the remainder had postgraduate diplomas (14%), MPhil/PhDs by thesis (14%), or doctorates (7%).

**Table 5:** PN adopters’ sector affiliation and academic qualifications.

Sector	Frequency	Percent
Public	7	50%
Private	5	36%
Non-profit/charity	2	14%
Qualification		
BSc	5	36%
Masters	4	29%
Postgraduate diploma	2	14%
PhD/MPhil by thesis	2	14%
Doctorate	1	7%

### Qualitative analysis

#### Perceived gaps and barriers in the adoption of PN practices in Saudi Arabia

The qualitative analysis of perceived barriers to the adoption of PN in Saudi Arabia revealed several recurring themes reflecting systemic, educational, and cultural challenges. As summarized in Table 6, the most frequently cited barrier was lack of awareness (*n* = 27), with many participants noting limited public understanding of PN and its distinction from conventional dietary advice. This lack of awareness extends to some healthcare professionals as well, which undermines trust, interest, and uptake of such innovations, examples of dietitians quotes; “Limited public awareness, insufficient access to qualified nutrition professionals, Lack of complete knowledge about nutrition Genomics and Microbiomes studies in KSA, Untried RD or lack of knowledge, Limited awareness, knowledge, research, and resources, Lack of research on this practice lack of training lack of updates on the latest practices.”

**Table 6:** Summary of perceived barriers to PN adoption.

Theme	*N*	Illustrative quotes
Lack of awareness	27	“Lack of awareness and understanding about PN.” “The community and some dietitians are not well-informed about these practices.” “Limited public awareness of health and disease prevention.”
Cost and accessibility	21	“High cost of testing and personalized meal planning is a big hurdle.” “Resources are limited and expensive.” “Cost, time consumed during consultations, and shortage of RDs.”
Technological infrastructure	19	“We lack the labs and equipment to run gene or microbiome analysis.” “Lack of high technological assessment tools like metabolic cart.” “Absence of essential lab tests in public hospitals.”
Training and education	17	“Lack of education in nutrition genomics in our curriculum.” “Limited training by the dietitians.” “Lack of updates on latest practices and training in new technologies.”
Healthcare system limitations	14	“Shortage of specialized healthcare providers who can follow up.” “Lack of integration within healthcare settings.” “No many dietitians employed across hospitals or schools.”
Cultural and social influences	10	“Cultural dietary habits are hard to change.” “Social pressure to consume large amounts of food during events.” “Traditional meals like Kabsa do not align with PN.”
Regulatory barriers	8	“There are no clear regulations for PN practices.” “Unclear clinical guidelines in specific areas of nutrition.” “Lack of standardization or unified practice policies.”
Research and evidence	7	“Very limited local research is available to support implementation.” “Lack of Saudi-specific data to back personalized approaches.” “Absence of baseline data on population nutrition genomics.”
Time constraints	4	“Consultation time is too short for personalized diet plans.” “Short sessions do not allow enough time to explain individualized plans.” “Healthcare providers struggle to thoroughly investigate individual needs due to time limits.”

Cost and accessibility (*n* = 21) emerged as another significant concern. Respondents emphasized the high costs associated with genetic and microbiome testing, along with personalized meal planning services, many of which are not covered by insurance or readily available in public health facilities “cost barriers, Lack of resources and time given to each patient during visits, high costs.” These constraints particularly affect lower-income groups and widen health disparities. Closely related is the barrier of technological infrastructure (*n* = 19), with several participants noting the scarcity of advanced laboratories, diagnostic tools, and digital platforms required to support PN services. This infrastructure gap impedes integration with mainstream healthcare systems.

Another critical theme was training and education (*n* = 17). Participants reported that existing nutrition and medical curricula offer little or no instruction in nutrigenomics or related emerging fields, leaving practitioners underprepared “Lack of education and knowledge about PN (PS), and there is no deep and specific curriculum to study PS.” Compounding this are healthcare system limitations (*n* = 14), such as the shortage of specialized providers and limited consultation time “Short sessions with patients that happens always in the hospitals since the dietitian will not have enough time to prepare, discuss and explain to the patient about his diet. In my hospital we used to have 30 min for each session with the patient and it helped a lot. Unfortunately we had to change the session time to 15 min due to the high demand, which hinder the delivery of PN care, comprehensive systems that allow for thorough assessments such as genomics testing and a variety of essential laboratory tests that might not be available at the majority of health centres including public hospitals.”

Cultural and social influences (*n* = 10) were also reported as significant barriers, including entrenched dietary habits, social pressure in communal eating settings, and general resistance to behaviour change. Regulatory barriers (*n* = 8) were noted by some participants, who pointed to the absence of clear national guidelines or licensing protocols, creating ambiguity for practitioners and businesses alike. Additionally, a lack of local research and evidence (*n* = 7) was cited as a challenge to gaining credibility and securing stakeholder support for PN interventions. Finally, time constraints during patient consultations (*n* = 4) were highlighted as an operational hurdle limiting meaningful patient engagement.

Together, these findings underscore the multifaceted nature of the barriers to PN adoption in Saudi Arabia—spanning infrastructure, workforce development, cultural norms, policy, and equity issues. Addressing these barriers will be essential to successfully integrating PN into the country’s healthcare landscape.

#### Key facilitators for implementing PN strategies in Saudi Arabia

The thematic analysis of participants’ responses revealed seven key facilitators that could support the implementation and uptake of PN approaches in Saudi Arabia. These facilitators are summarized in Table 7, organized by thematic categories and supported by illustrative quotes.

**Table 7:** Facilitators for implementing PN in Saudi Arabia.

Theme	*N*	Illustrative quotes
Education, awareness and training	19	“Provide workshops and certification… Include this in nutrition programs in universities.” Training the students and teach them more… workshops and class.” Raising awareness about the benefits of PN.”
Government, policy and institutional support	14	“The support of the government and ministry of health.” “Clear guidelines in clinical nutrition practices in Saudi Arabia need to be established…” “Developing policies and guidelines for standardized practice…”
Technology and innovation integration	8	“Introduce technology and using innovative methods…” “Use of AI, laws and workshops… translate nutrition ideas into tools and software.” “AI and technology-related practices and applications.”
Research, data and infrastructure	6	“More research on how our bodies respond to different foods and nutrients.” “Sufficient funding for R&D to collect comprehensive baseline data.” “Start to integrate these technologies with healthcare system.”
Access, affordability and inclusion	5	“Important nutritional analyses should be accessible to everyone…” “Include nutrition clinics in the insurance system.” “Affordability and cost-effectiveness.”
Collaboration and interdisciplinary practice	4	“Build confidence between RD and physicians.” “Collaboration with healthcare providers.” “Stakeholders integration.”
Cultural adaptation and behaviour change	3	“Consider traditional food… imagine the needed in society.” “Behavioral modification.” “Cultural integration… priority to quality over quantity.”

The most frequently cited facilitator (*n* = 19) was the need for enhanced education, awareness, and training. Participants emphasized the importance of integrating PN into university curricula, providing specialized workshops, and raising public awareness “Provide workshops and certification in PN. Include this in nutrition programs in universities.” Suggestions included offering certifications for dietitians, incorporating genomic and technological topics in nutrition programs, and increasing awareness about the benefits of PN among both professionals and the general public. The second most frequently mentioned facilitator (*n* = 14) was government, policy, and institutional support “The support of the government and ministry of health,” “comprehensive system that allows for thorough individual assessment.” Participants highlighted the crucial role of the Ministry of Health and other regulatory bodies in establishing clear policies, guidelines, and legal frameworks to regulate and standardize PN practices. Several emphasized the need for government-led initiatives to ensure alignment with national healthcare strategies and to provide credibility to PN services “Updated standards of care and guidelines in work settings.”

Technology and innovation integration (*n* = 8) was also regarded as an important enabler. Respondents advocated for the introduction and promotion of advanced technologies such as artificial intelligence, digital platforms, and software tools tailored for nutrition professionals “increasing AI and technology-related practices and applications among practitioners and researchers “. These tools were seen as essential for translating complex genetic and metabolic data into practical dietary recommendations. A related facilitator, cited by 6 participants, was the need to invest in research, data, and infrastructure. This theme encompassed calls for increased funding for research and development, the collection of localized health and nutrition data, and the integration of PN tools into existing healthcare systems “I feel that Saudi Arabia needs to put more attention and resources that supports implementation of PN. In this way, we can conduct more research and trials to test its feasibility and effectiveness and hopefully advance the field.”

Addressing access, affordability, and inclusion was mentioned by five participants. Respondents underscored the importance of ensuring that diagnostic tools and PN services are accessible to a broad segment of the population, including through insurance coverage and cost-effective service delivery models. Collaboration and interdisciplinary practice (*n* = 4) were highlighted as a means of strengthening the implementation process. Participants suggested fostering cooperation between registered dietitians, physicians, healthcare providers, and other stakeholders to ensure a more cohesive and efficient approach to personalized care.

Finally, cultural adaptation and behaviour change were noted by 3 participants as necessary factors for effective adoption. Respondents stressed the importance of tailoring recommendations to fit cultural food practices and encouraging behavioural changes that align with Saudi social norms and values. These facilitators reflect both systemic and individual-level strategies for promoting PN in Saudi Arabia. Their identification provides actionable insights for policymakers, educators, and healthcare professionals seeking to advance this emerging field.

#### Cultural and social critical areas for implementing PN tailored to the Saudi population

The survey participants highlighted several critical areas necessary for developing culturally tailored nutritional strategies in Saudi Arabia (Table 8). Through thematic analysis of their open-ended responses, seven core themes emerged, which are summarized in Table 7 along with their frequency and illustrative quotes.

**Table 8:** Summary of critical areas for developing culturally tailored nutritional strategies in Saudi Arabia.

Theme	*N*	Illustrative quotes
Education, awareness, and school-based interventions	18	“More awareness should be given to school age children…”- “Spreading the knowledge of the importance of PN…”- “School nutrition…”
Cultural and traditional dietary practices	10	“Incorporate cultural practices…”- “We need to integrate our cultural and traditional dietary habits…”- “Understand food habits and traditional food practices…”
PN and individual needs	8	“Importance of PN…”- “We need to know the impact of PN in Saudi Arabia…”- “Detailed evidence-based food analysis programs…”
Obesity and lifestyle-related health issues	7	“Addressing high rates of obesity and lifestyle-related diseases…”- “Obesity treatment and weight management…”- “Focus on reducing added sugar…”
Data collection and research infrastructure	6	“Profile a representative sample of the population…”- “Nutritional strategies start from data derived from research…”- “Food database based on Saudi food…”
Policy support and clinical guidelines	4	“Clear regulations, accuracy…”- “RDAs, Clinical guidelines for nutritional related disease…”- “Policy support and regulation…”
Community engagement and holistic wellbeing	4	“Medical corner at each event…”- “Promote a healthy environment…”- “Focus on mental health and stress…”

The most frequently mentioned theme was education, awareness, and school-based interventions, raised by 18 participants. Respondents emphasized the need to enhance public understanding of nutrition, starting from an early age, particularly through school-based programs and awareness campaigns. Illustrative comments included: “More awareness should be given to school-age children…” and “Spreading the knowledge of the importance of PN…” and.

The second most common theme, cited by 10 participants, was cultural and traditional dietary practices. This theme underscored the necessity of integrating culturally appropriate food habits and respecting traditional culinary practices in national dietary strategies. Participants stated: “We need to integrate our cultural and traditional dietary habits…” and “Understand food habits and traditional food practices…” and “‘strategies should aligned with traditional dietary preferences to make it easy to follow it.”

PN and Individual Needs was the third most frequent theme, referenced by 8 participants. Respondents called for the development of individualized dietary approaches based on scientific tools such as genetic profiling and personalized assessments. Comments included “Importance of PN…” and “Detailed evidence-based food analysis programs….”

Obesity and lifestyle-related health issues were raised by seven participants, reflecting growing concern over the prevalence of obesity, diabetes, and related conditions in the population. Participants recommended strategies aimed at sugar reduction, weight management, and promotion of physical activity, such as “Addressing high rates of obesity and lifestyle-related diseases….”

The need for data collection and research infrastructure was expressed by six participants, who highlighted the importance of evidence-based policymaking, local databases, and national nutritional surveys. For example, one participant noted: “Nutritional strategies start from data derived from research….”

Two themes were mentioned by four participants each: policy support and clinical guidelines, and community engagement and holistic wellbeing. The former focused on the need for regulatory frameworks, standardized dietary guidelines, and clear national recommendations (e.g., “RDAs, Clinical guidelines for nutrition-related disease…”), while the latter emphasized inclusive strategies for promoting physical and mental health at the community level, such as “medical corners at each event…” and “Focus on mental health and stress….”

These themes collectively highlight a multidimensional understanding among participants regarding the components needed to create effective, culturally sensitive nutrition strategies tailored to the Saudi population.

## 4 Discussion

The findings of this study indicate that Saudi Arabian registered dietitians are comfortable with the idea of PN but face significant challenges in putting it into practice. According to our data, only 16% of participants adopted PN (Table 1). This rate is similar to 15% adoption rate among a geographically diverse sample of registered dietitians (20). It’s evident that there’s a gap between professional enthusiasm for new technologies and the necessary practical infrastructure to make them successful (21, 22).

The adoption of PN in Saudi Arabia requires synchronized interventions at three interdependent levels. (1) Education: dietetics extracurricular and professional development courses and workshops in technology and omics literacy, nutrigenomics, metabolomics and microbiome counselling with focus on evidence based training. (2) Policy frameworks: enabling system-level implementation through guidelines from the Ministry of Health and hospitals, funding structures for PN education and services, and regulatory oversight of PN testing quality. (3) Infrastructure: a critical factor in practical feasibility, partnerships between laboratories and companies to analyse biomarkers, interoperable electronic health records to integrate data, and technology procurement (23–25).

The educational element covers competencies such as research skills for critical evaluation, PNT and assessment methods that enhance patient care in clinical practice, and evidence-based practice to support informed decision-making. Based on the strong correlation between PNT usefulness and research (*r* = 0.213, *p* = 0.043) as shown in (Table 4), Saudi dietitians who prioritize scientific validity are more likely to view PN as credible and promising. This quantitative finding was supported by qualitative narratives, where participants pointed to the foundational role of research frameworks highlighting a critical gap: research infrastructure is still lacking to support PNT implementation in Saudi Arabia. This finding is particularly encouraging as it emphasizes that PN adoption is being driven by scientific merit (26), which is consistent with reviews showing that evidence strength and quality are critical determinants of healthcare practice and innovation adoption (27, 28, 29).

In our qualitative findings dietitians revealed that enhanced education, awareness, and training were the leading facilitators for PN adoption (*n* = 19) (Table 7). These findings are consistent with previous research showing that educational gaps remain a significant barrier to PN implementation in clinical practice (30). To guide healthcare providers in incorporating nutrigenomics into clinical practice, the American Dietetic Association has developed a nutrigenomic care map as part of a great initiative (31). While there is an increasing demand for direct-to-consumer interpretation of nutrition genomic reports, practitioners currently lack clinical guidance documents. The initiative underscores the critical need for standardized protocols in PN implementation, especially given the big gap between basic research on diet-gene interactions and clinical practice. An important step toward bridging the theory-practice gap in PN is the development of care maps, protocols, and training on methodologies used for PN.

The current dietetic curriculum in Saudi Arabia emphasizes medical nutrition therapy (MNT), but provides limited training in omics interpretation, technology-assisted counselling, and precision assessment integration-highlighting the educational gaps identified as barriers to clinical PN adoption in our study. In MNT, PN should be an adjunctive clinical decision support practice rather than an independent intervention. When integrated within Saudi practice, PN -omics assessment tools supplement standard assessments for complex cases, metabolic disorders, obesity management, and therapeutic diet services. In addition, PN delivers personalised health recommendations for disease prevention by factoring in genetics, metabolomics, microbiome and behaviour to improve adherence to dietary advice. Organizational support and inter-professional collaboration are strong adoption predictors in addition to dietitian readiness. Currently, Saudi Arabia’s clinical nutrition practice uses standard assessment tools and evidence-based MNT protocols without multi-omics investigation.

Complementing research and evidence-based practice as a foundational educational need, dietitians also need technology training in genetic, microbiome and metabolomics testing interpretation, and digital counselling training. The survey results in (Table 4) reveal a nuanced perspective among registered dietitians regarding PN technologies. Dietitians demonstrate strong openness to technological innovation—with 85% agreeing that incorporating the latest technologies helps provide optimal patient service and 89% holding positive views toward emerging technologies like machine learning and artificial intelligence. This was reinforced by qualitative data, where participants recognized technology integration as critical, emphasizing the need for “increasing AI and technology-related practices and applications.” This supports dietitians’ openness to innovation extends beyond theoretical acceptance to visions for technology-enabled practice. The enthusiasm aligns with the broader evolution of artificial intelligence nutritionists from basic intelligent systems to sophisticated platforms that integrate advanced algorithms, smart sensors, and big data for real-time PN, as research shifts toward molecular-level behavioural models and multi-centre and multi-level approaches (32, 33).

There is a wide range of confidence among dietitians regarding PN testing methods. Established diagnostic tools receive strong support, with 74% endorsing food allergy testing for diet personalization. Emerging methodologies also gain substantial acceptance, with 70% supporting microbiome testing and 68% favouring metabolomics. However, genetic testing presents ethical complexities, with 41% expressing uncertainty about ethical dilemmas and opinions evenly split on direct-to-consumer availability (32% agree, 34% disagree, 34% neutral). This pattern indicates dietitians are most comfortable with clinically established testing while showing cautious optimism toward newer biomarker technologies (42, 43). The uncertainty surrounding genetic testing highlights the need for clearer professional guidelines to support the responsible implementation of these emerging technologies. This was revealed by qualitative findings suggesting an educational gap, with participants reporting minimal curricula training in nutrigenomics, Participants called for “Provide workshops and certification in PN. Include this in nutrition programs in universities” suggesting that formalized training is essential for advancing evidence-based practice. A Canadian study of 457 healthcare providers found that 82.1% had no experience offering nutritional genomics to clients, with the primary barrier being the perception that nutrigenetic testing is too complicated (84.1%), while those who did integrate testing were motivated by beliefs that clients would be more motivated to change eating habits (70.4%) (34).

These educational elements address individual-level readiness. The second implementation level involves organizational and governmental systems that enable or constrain innovation adoption in PN-practice settings (25). The outcome that just over half of participants (54.5%) perceive their organizations as supportive of creative and innovative practices reveals a significant implementation challenge that extends beyond individual attitudes (Figure 1). With 45.5% of respondents expressing neutrality or disagreement regarding organizational innovation support, this finding aligns with broader healthcare innovation literature identifying organizational readiness as a critical implementation barrier (35). This aligns with qualitative findings, where participants described government support as facilitators of PN “The support of the government and ministry of health” while an equal number cited system challenges as barriers, including limited consultation time and insufficient testing infrastructure at most health centres. This suggests that structural enablers, adequate infrastructure, time, and institutional frameworks are the primary facilitators of implementation. In a cross-sectional study of 446 employees done at King Fahad Medical City (KFMC), Riyadh, to explore organizational culture and its impact from employees’ perspectives, innovation emerged as the weakest cultural dimension, scoring only 3.3/5 (66%). Addressing innovation deficiencies is essential for enhancing organizational competitiveness and advancing healthcare delivery excellence (36). International literature has emphasized the need for clear governance frameworks to guide the ethical and effective implementation of PN services (30).

The third implementation level involves infrastructure, although our survey focused on individual and organizational readiness perceptions, the literature consistently identifies infrastructure—laboratory partnerships, IT systems, and national and international collaborative frameworks—as factors of innovation in healthcare success (22, 31). According to our qualitative analysis, implementing PN programs requires addressing additional interconnected barriers. In addition to cost and accessibility concerns (*n* = 21), technological infrastructure gaps (*n* = 19), deficiencies in training and education (*n* = 17), healthcare system limitations (*n* = 14), cultural and social influences (*n* = 10), regulatory gaps (*n* = 8), limited local research evidence (*n* = 7), and time constraints during consultations (*n* = 4), implementation challenges are complex. Based on these findings, successful adoption of PN requires addressing economic barriers, developing infrastructure, including advanced laboratories, educational and digital platform partnerships.

In Table 9, we summarize the CFIR based analysis across all five domains and provide targeted implementation recommendations emphasizing innovation (37). Although evidence-based PN aligns with Vision 2030, it faces complexity and standardization challenges. Training programs focused on PN tools, patient consultation skills, and innovation mindset development are essential for developing individual capabilities. To improve the inner setting, IT infrastructure investments are required, as well as the establishment of dedicated PN innovation units. The development of outer settings requires national innovation policies and public-private partnerships. A key component of progress will be the development of culturally relevant AI-driven PN models through cross-sector partnerships and the establishment of formal innovation consortia that will accelerate evidence-based adoption and technology transfer. According to these findings, targeted healthcare workforce development and policy alignment with Saudi Vision 2030 are essential. Experiential learning and mentorship models are fundamental for nutrition professionals to successfully adopt digital health strategies and integrate multi-omics training, all of which are crucial for effective implementation. For instance, hands-on workshops and structured mentorship programmes can provide practitioners with practical experience in using digital health platforms and guidance on interpreting complex multi-omics data, enabling them to apply these advanced tools confidently and effectively in clinical settings. In parallel, dietetic curriculums should include PN competencies, as well as regulatory frameworks, such as the Ministry of Health’s Regulatory Healthcare Sandbox launch in 2024 (38). While international trials like Food4Me (39), Predict (40) and Toronto Nutrigenomics study (41) confirm the efficacy of PN in improving dietary adherence, successful implementation in the Middle East requires culturally adapted frameworks that respect traditional dietary patterns, which can be addressed through AI-driven approaches integrating cultural aspects such as food tastes (13). The initial advancements in genomics, together with the readiness for learning of Saudi Arabia’s healthcare professionals, and the governmental and private sectors research and innovation initiatives puts Saudi Arabia in a unique position to lead PN inventions in the region.

**Table 9:** CFIR-based implementation analysis and recommendations for PN in Saudi Arabia based on registered dietitians’ perceptions, attitudes, and readiness regarding PN and multi-omics approaches in Saudi Arabia.

CFIR domain	Facilitators	Barriers	Authors implementation suggestions
Innovation	Evidence-based foundation of PN; alignment with Vision 2030; innovation potential	Perceived resource intensity; lack of standardized protocols and training	Initiate collaborative research to build AI-driven PN models involving academia, business, and technology sectors; develop standardized PN protocols
Individuals: roles and characteristics	Motivation; innovation openness	Inadequate training; capability deficits	Develop training programs on PN diagnostic tools, and patient consultation skills; adopt innovation mindset through continuous professional development
Inner setting	Organizational support (54.5%); public sector resources	IT/laboratory resource gaps; limited training programs	Invest in IT infrastructure and laboratory resources; establish PN innovation units in healthcare facilities
Outer setting	Public financing availability, innovation incentives	Policy gaps; infrastructure limitations	Develop national PN innovation policies aligned with Vision 2030; strengthen public-private partnerships
Implementation process	Research engagement among dietitians	Limited implementation protocols; lack of systematic adoption pathways	Establish systematic implementation protocols; create cross-sector innovation consortia for PN development and technology transfer

This study acknowledges both the small sample size and the low response rate, which may limit the generalizability of the findings beyond the study sample rather than the internal validity of the results. Another limitation concerns the validation of the qualitative component; first party validation was performed through feedback from four subject matter experts, who assessed the content relevance and clarity of both the quantitative and qualitative questionnaires. This expert feedback contributed to the study’s overall rigour by ensuring the appropriateness and comprehensibility of the survey instruments. Due to the limited number of participants, pilot testing was not conducted. The quantitative questions were adopted from a pre-validated scale. Small sample size and low response rate are common challenges in specialized nutrition research involving healthcare professionals and do not necessarily undermine the validity of the findings. However, the study’s strengths include the use of a mixed-methods approach that combines quantitative and qualitative data to provide comprehensive insights into PN readiness, barriers and facilitators, and the inclusion of participants from different regions across Saudi Arabia and different work sectors, which enhances contextual representativeness within the national setting, even though with limited external generalizability. It is important to note that these results apply to perceptions of readiness for implementation and do not provide evidence regarding actual patient health outcomes or intervention efficacy.

Future research should advance PN through pilot trials in metabolic and obesity clinics, evaluating patient outcomes, workflow, practitioner training, and cost-effectiveness compared to standard care for Ministry of Health policy decisions. Methods need to adapt PN to Saudi cultural norms, such as traditional diets and family-based eating. Infrastructure research efforts should develop big data and AI platforms that integrate multi-omics for Middle Eastern populations, addressing the current lack of Arab genomic representation which limits PN accuracy.

## 5 Conclusion

This study represents one of the first comprehensive investigations of PN attitudes among dietitians in the Middle East. In Saudi Arabia, adoption of PN has remained low, highlighting a significant knowledge-practice gap. Comprehensive training and education programs tailored to the Saudi healthcare context are essential to bridging this knowledge-practice gap. In spite of limited adoption, healthcare professionals recognize the importance and potential of PN approaches. The need for structured educational interventions, technology training, and culturally appropriate implementation frameworks is urgent in order to translate this enthusiasm into practical competencies. Currently, PN is evolving from a research concept into a clinical reality, and understanding professional perspectives in diverse healthcare settings will support its successful implementation.

## Data Availability

The raw data supporting the conclusions of this article will be made available by the authors, without undue reservation.
